# Psychometric properties of the Chinese version of the PROMIS-Cancer-Anxiety item bank assessed using a graded response model

**DOI:** 10.1016/j.apjon.2023.100312

**Published:** 2023-09-28

**Authors:** Tingting Zhou, Yiwei Wang, Jialin Chen, Qingmei Huang, Fulei Wu, Hao Zhang, Changrong Yuan, Tingting Cai

**Affiliations:** a School of Nursing, Fudan University, Shanghai, China; b Nursing Department, Shanghai Geriatric Medical Center, Shanghai, China

**Keywords:** Anxiety, Cancer, Patient-Reported Outcome Measurement Information System, Item response theory, Graded response model, Item bank

## Abstract

**Objective:**

This study aimed to examine the psychometric properties of the Chinese version of the Patient-Reported Outcome Measurement Information System (PROMIS)-Cancer-Anxiety item bank using a graded response model in a sample of patients with cancer.

**Methods:**

A cross-sectional study was conducted and the Chinese version of the PROMIS-Cancer-Anxiety item bank was used to measure anxiety in patients with cancer. The unidimensional structure of the item bank was evaluated using principal component analysis. Residual correlations and the graphs of item mean scores conditional on the rest scores were examined to evaluate the local independence and monotonicity of the items, respectively. Item characteristics were described using item parameter estimates and item information. Operating characteristic curves (OCCs) and test information curve (TIC) were also plotted. Measurement invariance across age, gender, and education level was assessed to identify possible differential item functioning (DIF).

**Results:**

A total of 1075 patients with cancer were enrolled. Under the assumptions of unidimensionality, local independence, and monotonicity, the discrimination parameters *a* ranged from 2.30 to 5.47, and the threshold parameters *b* ranged from *b*_*1*_ = −2.87 to *b*_*4*_ = 3.21 with proper intervals. Completely overlapped category curves were not observed among the OCCs of any items. Item information and TIC showed that the item bank had a wide measurement range. The DIFs for age, gender, and education level for all items were not remarkable.

**Conclusions:**

The results supported using the Chinese version of the PROMIS-Cancer-Anxiety item bank to measure anxiety and develop a computerized adaptive testing (CAT) system for anxiety in patients with cancer.

## Introduction

Cancer is the leading cause of morbidity and mortality worldwide. With improvements in screening, early detection, and treatment, many cancers are now defined as long-term conditions.^1^ Anxiety in patients with cancer is common, affecting approximately 10% to 19% during or after cancer treatment.^2^, ^3^, ^4^ As a multifactorial symptom, cancer-related anxiety decreases patient quality of life and influences treatment adherence.^5^^,^^6^ Evidence has also shown that clinically diagnosed anxiety disorders are always associated with increased cancer incidence, higher cancer-specific mortality, and poorer cancer survival.^7^ Accurate anxiety assessments are therefore necessary for patients with cancer.

The early assessment of anxiety has clinical and public health benefits for cancer prevention and treatment.^7^ It is recommended that all health care providers routinely assess the presence of emotional distress and specific anxiety symptoms from the point of diagnosis onward.^8^ The use of a valid and reliable tool for anxiety assessment with clinically meaningful, reportable scores is warranted; however, the abundance and heterogeneity of anxiety measures make it difficult to compare individual scales.^9^ Variations in item content and context, including timeframe, response scales, and the number of response categories, further complicate this task.^9^

The Patient-Reported Outcome Measurement Information System (PROMIS) solves these issues and is easily accessible with flexible administration and standardized scoring (www.nihpromis.org). The PROMIS-Cancer-Anxiety item bank (PROMIS-A) is a cancer-specific version of the PROMIS Chronic Illness banks that has undergone six rigorous development processes: domain identification and definition, qualitative item review, refinement of items for cancer populations, field testing, psychometric data analysis, and evaluation of item banks.^10^ To complete an efficient assessment of anxiety among patients with cancer using the PROMIS instruments, short forms and computerized adaptive testing (CAT) are the main administration options^11^; of the two, the CAT system has higher measurement efficiency and accuracy. To achieve this, using an item response theory (IRT)-calibrated item bank is key.^12^ In the measurement process, the CAT system chooses items from the bank that best match the person's level of latent trait (θ); it skips unnecessary items, enabling personalized testing for each patient and a more precise representation of the patient's trait.^12^^,^^13^ Because of this, constructing a high-quality item bank is crucial for effectively implementing and strengthening CAT systems.

IRT models the probability of selecting a particular item response category as a function of the person's level of θ and item characteristics and examines the performance of individual items of the scale and the appropriateness of response categories.^11^^,^^14^ The graded response model (GRM) proposed by Samejima is a commonly applied unidimensional IRT model applicable to Likert scale items.^15^ The GRM also has a wide range of applications with the PROMIS, as it provides guidance for forming PROMIS short forms and has profound implications for developing PROMIS CAT.^12^^,^^16^^,^^17^ For example, the Italian custom four-item short form selected from the PROMIS Anxiety Form 8a was formed based on analysis of the GRM,^17^ and the Dutch-Flemish version of the PROMIS Anxiety CAT was developed within the analytical framework of the GRM.^12^ Currently, the PROMIS-A has not been evaluated in Chinese patients with cancer, and a corresponding CAT system has not been developed. Thus, this study aimed to assess the psychometric properties of the PROMIS-A among patients with cancer under the guidance of the GRM.

## Methods

### Samples

Using convenience sampling, a cross-sectional study was conducted at Fudan University Shanghai Cancer Center and Fudan University Affiliated Zhongshan Hospital in Shanghai, Mainland China, from November 2020 to July 2021. Eligible patients clinically diagnosed with cancer, aged ≥ 18 years, knew their disease diagnosis and could speak and write in Mandarin were enrolled. Patients with cognitive deficits or critical medical conditions were excluded.

### Measures

#### The general information questionnaire

Demographic and clinical characteristics, including age, gender, education level, marital status, religious beliefs, long-term residence, employment status, cancer type, cancer metastasis, antineoplastic therapy, chronic diseases, and complications, were obtained with the general information questionnaire. The demographic characteristics were self-reported by the patients and the clinical variables were checked with the electronic medical records by trained nurse investigators.

#### The PROMIS-A

PROMIS-A is a cancer-specific version of the PROMIS anxiety item bank.^10^ Consisting of 23 items, the PROMIS-A assesses a wide range of anxiety-related feelings and symptoms within a 7-day recall period.^18^ Respondents were asked to rate how often they experienced these unpleasant feelings or symptoms on a five-point Likert scale (“Never” = 1, “Rarely” = 2, “Sometimes” = 3, “Often” = 4, “Always” = 5); a higher score indicated a higher level of anxiety. Authorization to translate the PROMIS-A into Chinese was obtained from the PROMIS Health Organization (PHO), and translation and cultural adaptation of the PROMIS-A was completed according to the Functional Assessment of Chronic Illness Therapy (FACIT) translation methodology.^19^ Related cognitive debriefing interviews for the PROMIS-A were conducted for patients with cancer in China to ensure the cultural equivalence of the instrument.^20^

### Data analysis

Descriptive statistics, including the demographic and clinical characteristics of the sample, as well as the distribution of PROMIS-A, were obtained using IBM SPSS version 26.0. Cronbach's α was considered an adequate internal consistency measure, with a criterion higher than 0.70 indicating good internal consistency.^21^ The homogeneity of the PROMIS-A was testified if Cronbach's α had no significant increase when the item was removed and when the correlation coefficients of the item-scale were higher than 0.40.^22^

Three main assumptions of GRM, including unidimensionality, local independence, and monotonicity, were evaluated in this study. Except for the unidimensionality, the other two assumptions were implemented using R version 4.2.2. The principal component analysis was used to examine the unidimensional structure of the instrument, with the premise that the statistic of the Kaiser-Meyer-Olkin test was close to 1 and the *P*-value of Bartlett's test of sphericity was less than 0.05; the assumption of unidimensionality was acceptable if the ratio of the first component characteristic root to the second component characteristic root exceeded three.^23^^,^^24^ The local independence of the items was verified with residual correlations lower than 0.70, and the monotonicity was supported with the graphs of item mean scores conditional on the rest scores (ie, total raw score minus the item scores).^25^^,^^26^

With the guidance of GRM, the psychometric evaluation of the PROMIS-A, including model fit, item parameter estimates, operating characteristic curves (OCCs), item information, test information curve (TIC), and differential item functioning (DIF), was conducted using R. The model fit was assessed by Root Means Square Error of Approximation (RMSEA), Comparative Fit Index (CFI), and Tucker–Lewis Index (TLI).^27^ RMSEA lower than 0.06 and CFI and TLI higher than 0.95 were used as the criterion for a good fit.^28^ The item parameter estimates of the PROMIS-A was conducted using the discrimination parameter *a* and the four threshold parameters *b*_*1*_ to *b*_*4*_. The discrimination parameter *a* indicates the extent to which the item could differentiate persons with similar θ estimates, and the four threshold parameters *b*_*1*_ to *b*_*4*_ show the value of θ at which a person had equal probabilities of choosing a higher over a lower response.^11^^,^^14^ When the value of the *a*-parameter was lower than 0.50, the item was assumed to be non-discriminating. When the absolute value of the *b*-parameter was higher than 10, the item was considered inappropriate for the respondent.^14^ The four threshold parameters, *b*_*1*_ to *b*_*4*_, should gradually be increased at intervals between 0.81 and 5 to show that the difficulty settings for adjacent response categories were reasonable.^29^ The OCCs were plotted to further illustrate the relationships between θ level and item response probability.^14^ The maximum information for each item was calculated, and the item information corresponding to a medium level of anxiety where the person's level of θ was 0 was provided. The TIC was depicted to demonstrate the test information that the PROMIS-A can provide for respondents with different anxiety levels.^14^ To assess whether an item had significant systematic errors due to group bias, both uniform DIF and non-uniform DIF were assessed for age (18–59 years and ≥ 60 years), gender (male and female), and education level (junior high school or below and high school or above). Ordinal logistic regression (OLR) methods were used to identify the presence of the DIF.^30^ To quantify the magnitude of DIF, the change in McFadden's pseudo R^2^ higher than 0.02 was set as the critical value for rejecting the hypothesis of no DIF.^30^

### Ethical considerations

Ethical approval for this study was provided by the institutional review boards of Fudan University Shanghai Cancer Center (IRB No. 1810192-22) and Fudan University Affiliated Zhongshan Hospital (IRB No. 2020-076R). Trained researchers invited eligible patients to participate. The patients were informed about the aims and procedures of the study and the voluntary nature of participation. Patients signed informed consent forms and completed the investigation using paper-based questionnaires. While filling in the questionnaires, the researchers answered any questions raised by the participants if necessary and provided explanations for items without any inducement. After all questionnaires were completed, their integrity was checked.

## Results

### Descriptive statistics

A total of 1100 questionnaires were sent out, and 1075 were completed. The effective recovery rate was 97.73%. The demographic and clinical characteristics of the study population are shown in Table 1. The mean age of the population was 53.62 years [standard deviation (SD) = 13.48, range: 24–87 years]. Most patients were female (62.0%) and had been diagnosed with gynecological cancer (43.2%). The average PROMIS-A score was 69.55 (SD = 16.53, range: 36–108). The PROMIS-A also showed a high internal consistency reliability (Cronbach's α = 0.98), which remained unchanged regardless of which item was omitted from the item bank. Convergent validity was acceptable, with correlation coefficients between items and the item bank greater than 0.40 (range: 0.75–0.89, *P* < 0.05).

**Table 1:** Demographic and clinical characteristics of the study population (*N* = 1075).

Characteristics	Frequency (Percent, %)
Age	
18–59 years	693 (64.5)
≥ 60 years	382 (35.5)
Gender	
Male	408 (38.0)
Female	667 (62.0)
Education level	
Junior high school or below	505 (47.0)
High school or above	570 (53.0)
Marital status	
Married	960 (89.3)
Unmarried/divorced/widowed	115 (10.7)
Religious belief	
Yes	346 (32.2)
No	729 (67.8)
Long-term residence	
Rural area	287 (26.7)
Urban area	788 (73.3)
Employment status	
Unemployed	270 (25.1)
In service	534 (49.7)
Retired	271 (25.2)
Cancer type	
Gynecological cancer	464 (43.2)
Liver cancer	298 (27.7)
Colorectal cancer	149 (13.9)
Gastric cancer	105 (9.8)
Breast cancer	59 (5.5)
Cancer metastasis	
Yes	295 (27.4)
No	289 (26.9)
Unclear	491 (45.7)
Antineoplastic therapy	
Yes	489 (45.5)
No	586 (54.5)
Chronic disease	
Yes	602 (56.0)
No	473 (44.0)
Complication	
Yes	214 (19.9)
No	861 (80.1)

### Assumptions for the GRM

First, the statistic of the Kaiser-Meyer-Olkin test was 0.962, and the *P*-value of Bartlett's test of sphericity was lower than 0.001. The ratio of the first component characteristic root to the second component characteristic root was 13.11, indicating that the PROMIS-A conformed to the assumption of unidimensionality. Second, all item pairs were assumed to be locally independent, with residual correlations ranging from −0.40 to 0.62. Thirdly, the assumption of monotonicity was established with the graphs of item mean scores conditional on the rest scores in all items showing a gradually increasing trend.

### Model fit and item parameter estimates

The model fit indices indicated a good fit of the GRM (RMSEA = 0.05, TLI = 0.97 and CLI = 0.96). The results of the PROMIS-A item parameter estimates are shown in Table 2. The discrimination parameter *a* was higher than 0.50, ranging from *a* = 2.28 to *a* = 5.47. The threshold parameter *b* was also in an acceptable range, ranging from *b*_*1*_ = −2.94 to *b*_*4*_ = 3.21. In addition, all four item threshold parameters gradually increased. Except for the interval between *b*_*1*_ and *b*_*2*_ in the item EDANX09, which was slightly lower than 0.81, all the other intervals of the *b*-parameters in the items were between 0.81 and 5.

**Table 2:** Item parameter estimates of the PROMIS-A.

Items	*a*	*b*_*1*_	*b*_*2*_	*b*_*3*_	*b*_*4*_	*b*_*2*_*-b*_*1*_	*b*_*3*_*-b*_*2*_	*b*_*4*_*-b*_*3*_
EDANX27: I felt something awful would happen.	4.05	−2.52	−1.12	0.12	1.21	1.40	1.24	1.09
EDANX53: I felt uneasy.	5.16	−2.33	−1.13	0.06	1.34	1.20	1.19	1.28
EDANX05: I felt anxious.	5.47	−2.29	−1.17	0.05	1.46	1.12	1.22	1.41
EDANX12: I felt upset.	4.13	−2.68	−0.84	0.33	1.66	1.84	1.17	1.33
EDANX55: I had difficulty calming down.	3.88	−2.31	−0.50	0.71	1.95	1.81	1.21	1.24
EDANX01: I felt fearly.	4.90	−1.63	−0.30	0.93	2.01	1.33	1.23	1.08
EDANX02: I felt frightened.	4.08	−1.75	−0.27	1.04	2.07	1.48	1.31	1.03
EDANX33: I felt terrified.	4.37	−1.60	0.04	1.29	2.13	1.64	1.25	0.84
EDANX08: I was concerned about my mental health.	2.68	−1.55	0.10	1.58	2.63	1.65	1.48	1.05
EDANX47: I felt indecisive.	2.28	−2.01	−0.54	1.06	3.21	1.47	1.60	2.15
EDANX18: I had sudden feelings of panic.	2.76	−1.81	−0.37	1.06	2.66	1.44	1.43	1.60
EDANX26: I felt fidgety.	2.86	−1.92	−0.42	0.98	2.58	1.50	1.40	1.60
EDANX07: I felt like I need help for my anxiety.	2.62	−1.48	0.37	1.63	2.62	1.85	1.26	0.99
EDANX30: I felt worried.	2.60	−2.87	−1.48	0.10	1.66	1.39	1.58	1.56
EDANX46: I felt nervous.	3.39	−2.48	−1.52	0.00	1.38	0.96	1.52	1.38
EDANX51: I had trouble relaxing.	3.66	−2.08	−0.98	0.32	1.67	1.10	1.30	1.35
EDANX54: I felt tense.	2.87	−1.98	−0.49	0.79	2.30	1.49	1.28	1.51
EDANX41: My worries overwhelmed me.	2.69	−1.72	0.24	1.39	2.66	1.96	1.15	1.27
EDANX03: It scared me when I felt nervous.	2.30	−2.43	−0.49	0.89	2.23	1.94	1.38	1.34
EDANX48: Many situations made me worry.	3.32	−2.57	−1.59	−0.22	1.28	0.98	1.37	1.50
EDANX09: I had unpleasant thoughts that wouldn't leave my mind.	4.23	−2.42	−1.68	−0.33	1.13	0.74	1.35	1.46
EDANX39: I worried about dying.	2.37	−2.94	−1.67	−0.23	0.88	1.27	1.44	1.11
EDANX40: I found it hard to focus on anything other than my anxiety.	2.70	−1.55	0.22	1.57	3.21	1.77	1.35	1.64

PROMIS-A, PROMIS-Cancer-Anxiety item bank.

### The OCCs of the PROMIS-A

**Fig. 1:**
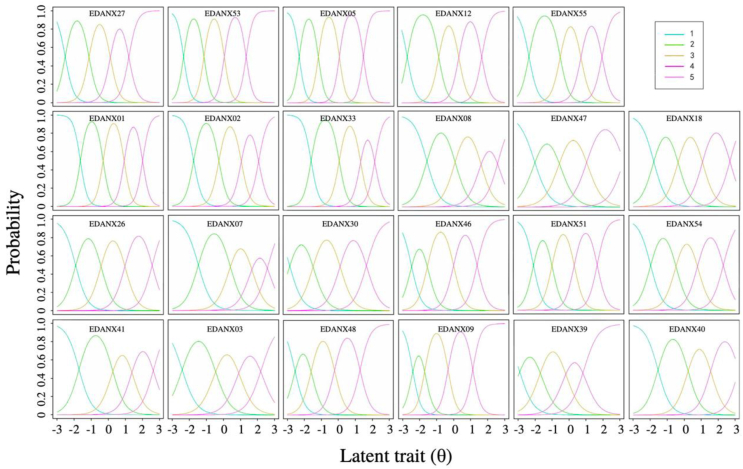
The operating characteristic curves (OCCs) of the PROMIS-Cancer-Anxiety item bank (PROMIS-A).

The probabilities of respondents with different levels of θ choosing any category scored from 1 to 5 are shown in Fig. 1. Each line represents one response category on a five-point Likert-type response. The x-axis represents different levels of θ. The y-axis represents the probability of selecting the response category. For all the items of the PROMIS-A, the category curves of any single item were not entirely overlapped by other curves, indicating that each category in the items played a proper role in the measurement.

### Item information

The maximum amount of information provided by each item ranged from 1.38 to 7.49 (Table 3). Ten out of the twenty-three items had maximum information higher than 3.0, and the level of θ ranged between −2.3 and 2.0. In addition, the item information when the person's level of θ was close to the corresponding maximum information indicated that the PROMIS-A was suitable for testing cancer patients with medium anxiety levels.

**Table 3:** Item information of the PROMIS-A.

Item code	Maximum information (corresponding θ)	Information (θ = 0)
EDANX27	4.15 (0.1)	3.92
EDANX53	6.64 (−2.3)	6.53
EDANX05	7.49 (−2.3)	7.33
EDANX12	4.31 (0.3)	3.09
EDANX55	3.83 (0.7)	2.30
EDANX01	6.03 (2.0)	3.81
EDANX02	4.22 (1.1)	3.26
EDANX33	4.92 (1.3)	4.75
EDANX08	1.93 (1.7)	1.82
EDANX47	1.38 (−0.6)	1.14
EDANX18	1.97 (−0.4)	1.68
EDANX26	2.11 (−0.4)	1.70
EDANX07	1.88 (1.7)	1.44
EDANX30	1.76 (−1.5)	1.72
EDANX46	2.98 (−1.6)	2.91
EDANX51	3.44 (−1.0)	2.61
EDANX54	2.14 (−0.5)	1.75
EDANX41	1.94 (1.4)	1.69
EDANX03	1.42 (0.9)	1.27
EDANX48	2.89 (−1.6)	2.50
EDANX09	4.67 (−1.7)	2.96
EDANX39	1.54 (−0.1)	1.53
EDANX40	1.88 (1.5)	1.73

PROMIS-A, PROMIS-Cancer-Anxiety item bank.

### The TIC of the PROMIS-A

**Fig. 2:**
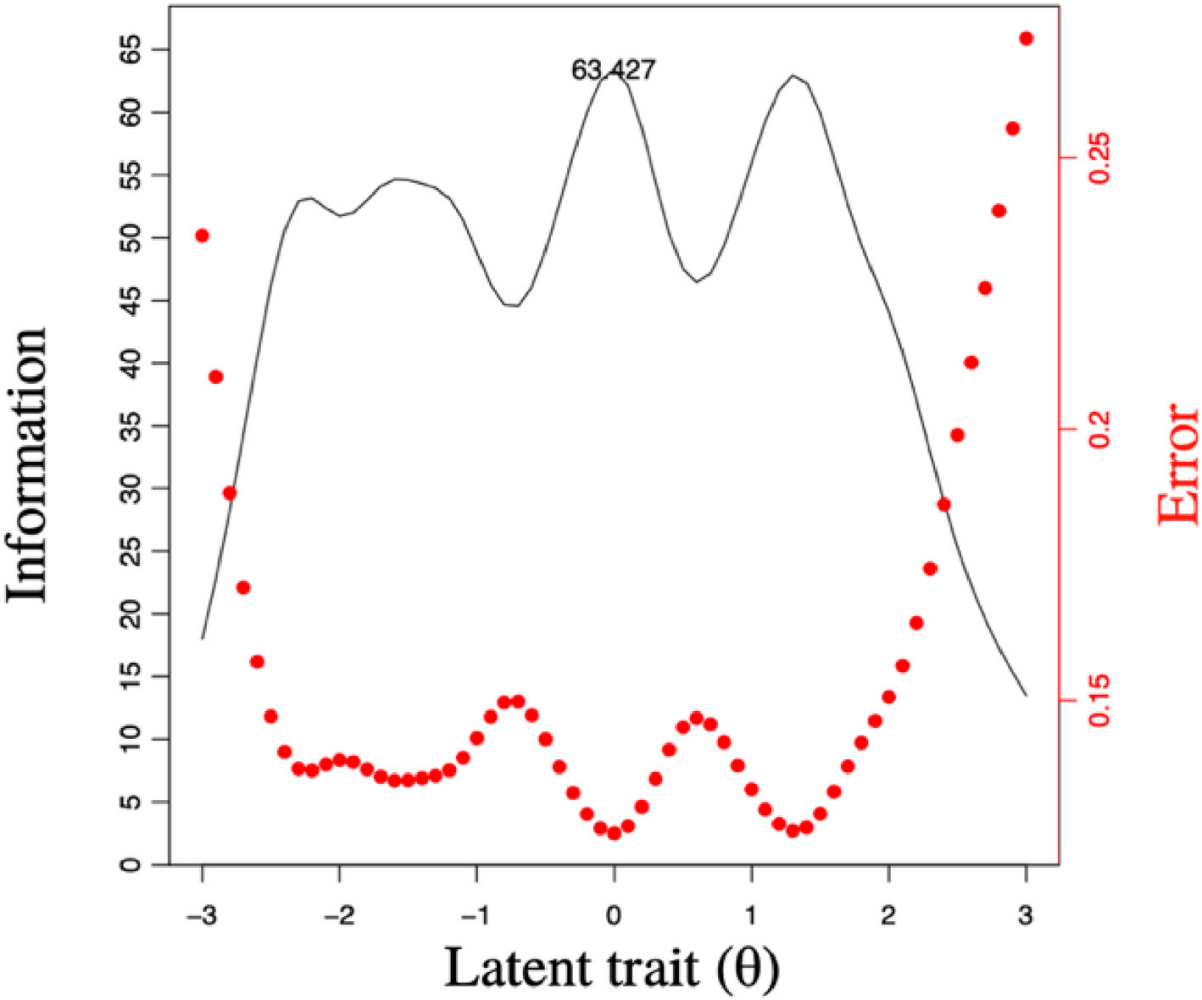
The test information curve (TIC) of the PROMIS-Cancer-Anxiety item bank (PROMIS-A).

As shown in Fig. 2, the test information varied with the θ level. The curve indicated that when the level of θ was around 0, the test information reached its highest value of 63.427. In addition, the PROMIS-A was highly informative within a wide range of anxiety levels, with θ between −3.0 and 3.0. The test information rapidly decreased when θ exceeded the limit by −2.5 to 1.5.

### DIF

Uniform and non-uniform DIF were present in PROMIS-A. Five items (EDANX27, EDANX01, EDANX33, EDANX46, and EDANX09) were identified as having a uniform DIF concerning age. Three items (EDANX05, EDANX51, and EDANX54) were identified for uniform DIF concerning gender, and one item (EDANX02) was identified for non-uniform DIF. Additionally, EDANX55 exhibited uniform DIF for the education domain, and EDANX27 exhibited non-uniform DIF. However, the combined impact was negligible for all DIF variables, with McFadden's pseudo R^2^ lower than 0.02.

## Discussion

Studies focusing on testing anxiety item banks are important for promoting the efficient application of patient-reported outcome measurements (PROMs).^12^^,^^31^ To the best of our knowledge, this is the first study to evaluate the psychometric properties of the Chinese version of the PROMIS-A using GRM in a sample of individuals with cancer.

The items from the PROMIS-A had a good threshold. All items in the PROMIS-A showed high discrimination, indicating that this scale can effectively distinguish and identify different levels of anxiety among patients with cancer. Compared to the Brazilian (discrimination range of 1.10 to 2.47), Dutch-Flemish (discrimination range of 1.34 to 3.59), and American (discrimination range of 1.26 to 3.86) versions of the PROMIS-Anxiety item bank, the Chinese version of the PROMIS-A showed a higher discrimination range (2.28 to 5.47) when applied to a population of patients with cancer.^12^^,^^32^ This discrepancy might be attributed to the cultural difference and the focused application of PROMIS-A in patients with cancer. Significant differences in patient-reported health measured with PROMIS profile 29 were also observed in the UK, France, and Germany.^33^ The level of item discrimination was reflected in the corresponding OCCs. Lower discrimination led to flatter OCCs, while higher discrimination gave steeper OCCs.^14^^,^^34^ All items showed an appropriate threshold range, with no cases in which the threshold parameters were particularly high or low; however, the item EDANX09 (*I had unpleasant thoughts that wouldn't leave my mind*) had a *b*_*1*_ and *b*_*2*_ interval of 0.74, slightly lower than 0.81, indicating that the difference between “rarely” and “sometimes” in the response category for this item was small. Similar results were found in the Brazilian and American versions of the PROMIS-Anxiety item bank, with several items having *b*_*1*_ and *b*_*2*_ intervals lower than 0.81.^12^^,^^32^ Duan et al^35^ claimed that it is difficult for individuals to make clear judgments about feelings, such as unpleasant thoughts; nevertheless, the threshold parameter intervals for other items in PROMIS-A were within an appropriate range. Considering the need to ease the measurement burden on patients, the wording of the response categories was kept consistent within the item bank.^16^

The value of information reflects the amount of information an item can provide when estimating a person's level of θ.^36^ Here, the maximum information for all items in the PROMIS-A was calculated along with the corresponding information for items when a person's level of θ was 0. Ten out of the twenty-three items had maximum information higher than 3.0, and the level of θ ranged between −2.3 and 2.0; this indicated that these items captured general levels of anxiety and that the PROMIS-A was suitable for measuring patients with cancer exhibiting medium levels of anxiety. Moreover, item information is closely related to the discrimination and threshold parameters, and items with higher discrimination values generally provide more information about the underlying θ.^37^ In this study, the item EDANX05 (*I felt anxious*) showed the highest score of 7.49, corresponding to the highest discrimination parameter of 5.47; the item EDANX47 (*I felt indecisive*) showed the lowest score of 1.38, corresponding to the lowest discrimination parameter of 2.28. The low-difficulty item was expected to perform poorly in differentiating between respondents at the high end of θ because they could easily meet the condition and scored high on the item, while the higher-difficulty item was expected to perform poorly in differentiating between respondents at the low end of θ because it was difficult for them to meet the condition. The spread of item information and the location on the θ scale at which the item information reaches its maximum can be determined by the category threshold parameters.^37^ Previous studies that used IRT to conduct personalized analyses of scale items recommended that items with more information be selected to create shorter measurement tools that may perform nearly as well as the original longer tools.^37^^,^^38^ The TIC depicted in this study further represents the correlation between the level of test information and the measurement precision, as greater amounts of test information led to smaller standard errors in measurement. Consistent with other language versions, the Chinese version of the PROMIS-A applied to patients with cancer also provided high amounts of test information across a wide range of anxiety levels.^9^^,^^39^

Test fairness is critical for the validity of group comparisons involving different characteristics, and DIF analysis can help ensure test fairness. In this study, both uniform and non-uniform DIF for age, gender, and education level were detected in the Chinese version of the PROMIS-A among patients with cancer. Consistent with the study by de Castro et al,^32^ statistically significant DIF for age and gender have previously been detected in the Brazilian version of the PROMIS-Anxiety item bank. The age-related DIF of the PROMIS-Anxiety item bank for American people was also detected.^30^ Although DIF items have appeared in different versions of the PROMIS-Anxiety item bank, the magnitude of the DIF was low in most cases. Furthermore, Flens et al^12^ reported that the Dutch-Flemish version of the PROMIS-Anxiety item bank presented no DIF items for age, gender, and education level, and all the items were enrolled in the subsequent CAT simulation.

Four schemes have been proposed for items showing DIF: deleting, ignoring, multiple-group modeling, and modeling DIF as a secondary dimension.^40^ However, the method of handling items exhibited in DIF remains unclear. Liu et al^41^ compared these four methods and suggested that different treatments should be used for different assessment purposes. Ignoring DIF items is the priority if the magnitude of DIF is small, whereas item parameter calibration is of the greatest interest for a short test.^40^^,^^41^ Additionally, the detection of DIF may be influenced by several factors, such as sparse data and small sample sizes.^42^ The combined impact of difference would always be negligible when weighted by a small number of the focal group trait distribution.^30^ Because of this, the treatment of DIF should be approached with caution for practical purposes.^43^

This study has several limitations. Firstly, the samples were collected using convenience sampling at two tertiary hospitals in Shanghai. Failure to recruit patients with cancer from other areas of China may have impaired the representativeness of the sample population. The PROMIS-A was also scaled such that the US general population had a mean score of 50 with an SD of 10.^16^ However, this study did not investigate cross-cultural validity and the available reference scores. DIF detection for language and linear transformation for calibrated scores could be adopted for a standard measurement of anxiety and horizontal comparisons between countries in the future. In addition, measuring anxiety in the real world is relatively complex, and multiple factors might have influenced the measurement process. Testing the item bank in combination with other advanced models is necessary to ensure accurate assessment.

## Conclusions

This psychometric investigation supported using the Chinese version of the PROMIS-A to measure moderate anxiety levels in patients with cancer. Using the GRM, sufficient psychometric information was obtained on individual items in the PROMIS-A, which enabled the PROMIS-A metric to be used while maintaining comparability with previous studies. The Chinese version of the PROMIS-A was found to be a suitable question source for the future development of a CAT system for patients with cancer.

## Acknowledgements

The authors thank and appreciate all patients participating in this study.

## CRediT author statement

**Tingting Zhou**: Conceptualization, Methodology, Data curation, Formal analysis, Writing. **Yiwei Wang**: Conceptualization, Methodology, Formal analysis, Writing – original draft preparation. **Jialin Chen**: Conceptualization, Methodology, Writing – review & editing. **Qingmei Huang**: Conceptualization, Methodology, Data curation. **Fulei Wu**: Conceptualization, Methodology, Data curation. **Hao Zhang**: Methodology, Data collection. **Changrong Yuan**: Conceptualization, Methodology, Data curation, Supervision, Writing – review & editing. **Tingting Cai**: Conceptualization, Methodology, Funding acquisition, Writing – review & editing. All authors had full access to all the data in the study, and the corresponding authors had final responsibility for the decision to submit for publication. The corresponding authors attest that all listed authors meet authorship criteria and that no others meeting the criteria have been omitted.

## Declaration of competing interest

The authors declare no conflict of interest. The corresponding author, Dr. Tingting Cai, is a member of the editorial board of the Asia-Pacific Journal of Oncology Nursing. The article underwent the journal's standard review procedures, with peer review conducted independently of Dr. Cai and their research groups.

## Funding

This research was supported by the Shanghai “Science and Technology Innovation Action Plan” soft science research project (Grant No. 23692114400) and the Fuxing Nursing Research Foundation of 10.13039/501100003347Fudan University (Grant No. FNF202355). The funding assisted in the language editing service of the manuscript. The funders had no role in considering the study design or in the collection, analysis, interpretation of data, writing of the report, or decision to submit the article for publication.

## Ethics statement

The study was approved by the Institutional Review Boards of Fudan University Shanghai Cancer Center (IRB No. 1810192-22) and Fudan University Affiliated Zhongshan Hospital (IRB No. 2020-076R). All participants provided written informed consent.

## Data availability statement

The data that support the findings of this study are available on request from the corresponding authors. The data are not publicly available due to their containing information that could compromise the privacy of research participants.

## Declaration of Generative AI and AI-assisted technologies in the writing process

No AI tools/services were used during the preparation of this work.
